# Performance of large language models for ophthalmic literature retrieval

**DOI:** 10.1038/s41433-026-04586-y

**Published:** 2026-06-12

**Authors:** Jai Paris, Oliver Kleinig, Ayushi Agarwal, Weng Onn Chan, Dinesh Selva

**Affiliations:** 1 Faculty of Health and Medical Sciences, Adelaide University, Adelaide, SA Australia; 2 South Australian Institute of Ophthalmology, Royal Adelaide Hospital, Adelaide, SA Australia; 3 Discipline of Ophthalmology and Vision Sciences, Adelaide University, Adelaide, SA Australia

**Keywords:** Outcomes research, Scientific community

Large language models (LLMs) such as ChatGPT, are increasingly used to search the literature and answer focused clinical questions. However, evidence on their reliability for study retrieval remains limited, particularly in Ophthalmology, where conditions are rare, terminology is nuanced, and niche management continually evolves [1]. Reports of hallucinated references in earlier-generation models underscores the need for contemporary, domain-specific evaluation [2].

We evaluated several widely accessible LLMs using five completed reviews as the reference standard. Each review was conducted using standard database search and selection methods, with included studies forming the “ground truth” corpus. Topics were heterogeneous, including surgical technique (glued amniotic membrane transplantation), orbital disease (sickle cell orbitopathy, orbital linear scleroderma), rare tumours (perivascular epithelioid cell tumour), and a risk factor review (retinal breaks during posterior vitreous detachment) [3, 4]. A concise natural language prompt was designed to reflect real-world clinician questioning (e.g., “Retrieve the literature on [topic]. Your output should include all publications”). Models evaluated included ChatGPT 5.2 (Auto and Deep Research), Claude Sonnet 4.6, Gemini 3 Pro, and Grok 4.1 thinking, all with default settings. Recall, precision, and F1 score were assessed across models and topics (Table 1). Differences were assessed using the Friedman test, with Wilcoxon signed-rank tests and Holm correction.

**Table 1:** Article retrieval performance by large-language models (LLMs) and the review article type.

Review topic, (article number)	LLM	Articles retrieved (*n*)	Irrelevant/incorrect (*n*)	Recall	Precision	F1 score
Sickle cell orbitopathy	ChatGPT 5.2 Auto	11	0	0.20	1.00	0.34
(*N* = 54 articles)	Grok 4.1 Thinking	8	0	0.15	1.00	0.26
	ChatGPT 5.2 Deep Research	13	0	0.24	1.00	0.39
	Claude Sonnet 4.6	5	0	0.09	1.00	0.17
	Gemini 3 Pro	3	1	0.06	0.75	0.10
Linear scleroderma orbitopathy	ChatGPT 5.2 Auto	7	0	0.37	1.00	0.54
(*N* = 19 articles)	Grok 4.1 Thinking	4	0	0.21	1.00	0.35
	ChatGPT 5.2 Deep Research	11	0	0.58	1.00	0.73
	Claude Sonnet 4.6	7	10	0.37	0.41	0.39
	Gemini 3 Pro	2	2	0.11	0.50	0.17
Orbital PEComa	ChatGPT 5.2 Auto	13	0	0.76	1.00	0.87
(*N* = 17 articles)	Grok 4.1 Thinking	5	0	0.29	1.00	0.45
	ChatGPT 5.2 Deep Research	11	0	0.65	1.00	0.79
	Claude Sonnet 4.6	9	1	0.53	0.9	0.67
	Gemini 3 Pro	5	1	0.29	0.83	0.43
Amniotic membrane transplant – acute SJS/TEN	ChatGPT 5.2 Auto	10	0	0.37	1.00	0.54
(*N* = 27 articles)	Grok 4.1 Thinking	6	0	0.22	1.00	0.36
	ChatGPT 5.2 Deep Research	12	0	0.44	1.00	0.62
	Claude Sonnet 4.6	6	2	0.22	0.75	0.34
	Gemini 3 Pro	2	0	0.07	1	0.14
Retinal break risk – PVD induction	ChatGPT 5.2 Auto	4	0	0.21	1.00	0.35
(*N* = 19 articles)	Grok 4.1 Thinking	2	1	0.11	0.67	0.18
	ChatGPT 5.2 Deep Research	3	0	0.16	1.00	0.27
	Claude Sonnet 4.6	5	1	0.26	0.83	0.40
	Gemini 3 Pro	5	1	0.26	0.83	0.40

*PEComa* perivascular epithelioid carcinoma, *SJS* Stevens-Johnson syndrome, *TENS* toxic epidermal necrolysis syndrome, *PVD* posterior vitreous detachment.

We found several interesting key findings. LLMs demonstrated low recall (0.16–0.41) but high precision (0.78–1.00), with F1 scores of 0.25–0.56. In practical terms, clinicians can be reasonably confident that LLM-generated lists are relevant, but many suitable studies are missed. ChatGPT Deep Research achieved the highest mean recall (0.41) and F1 score (0.56), while GPT Auto as well as Deep Research achieved perfect precision across all topics (1.00) (Table 2). Overall differences in performance were observed between models for recall (Friedman χ²(4) = 9.79, *p* = 0.044), precision (χ²(4) = 10.46, *p* = 0.033), and F1 score (χ²(4) = 9.66, *p* = 0.047), although no pairwise comparisons remained significant after correction (Table 2).

**Table 2:** Mean performance across topics and Friedman test with post hoc Wilcoxon signed-rank testing and Holm correction.

Model	Mean recall	Mean precision	Mean F1
ChatGPT 5.2 Auto	0.38	1.00	0.53
Grok 4.1 Thinking	0.20	0.93	0.32
ChatGPT 5.2 Deep Research	0.41	1.00	0.56
Claude Sonnet 4.6	0.29	0.78	0.39
Gemini 3 Pro	0.16	0.78	0.25
Omnibus and post hoc statistical tests			
Friedman χ²(4)	9.79	10.46	9.66
*p*-value	**0.044**	**0.033**	**0.047**
Wilcoxon testing + Holm correction	None significant	None significant	None significant

Bold values indicate *p* < 0.05.

Performance varied by topic, with higher recall for rarer topics (0.29–0.76) and lower recall for broader studies (0.06–0.24), likely reflecting limits on output length. LLM-based searches did not identify additional studies beyond those found by manual search, suggesting limited value as a search adjunct. Restricted access to paywalled content may also contribute to low recall.

Notably, hallucinated citations were essentially absent. The few non-relevant articles were better characterised as scope drift (for example, an article on ocular complications of systemic sclerosis instead of linear scleroderma of the orbit) rather than outright invention of journals, authors, or publication details (Table 1). This contrasts with earlier reports and suggests that, at least for the models and timeframe tested, reference generation may now be more reliable [5].

Our findings indicate that LLMs are best suited as high-precision tools for rapid literature scope, assisting clinicians quickly orientate themselves within an unfamiliar evidence base. However, they remain inadequate for comprehensive study identification in systematic reviews or guideline development. These findings are exploratory and likely limited by small sample sizes. As LLMs become more integrated into daily clinical practice, clinicians should recognise their potential while remaining cautious of their limitations.

## Data Availability

The datasets generated during and/or analysed during the current study are available from the corresponding author on reasonable request.
